# Beneficial Effects of *Ocimum gratissimum* Aqueous Extract on Rats with CCl_**4**_-Induced Acute Liver Injury

**DOI:** 10.1155/2012/736752

**Published:** 2012-06-27

**Authors:** Chun-Ching Chiu, Chih-Yang Huang, Tzy-Yen Chen, Shao-Hsuan Kao, Jer-Yuh Liu, Yi-Wen Wang, Bor-Show Tzang, Tsai-Ching Hsu

**Affiliations:** ^1^Institute of Microbiology and Immunology, Chung Shan Medical University, Taichung 40201, Taiwan; ^2^Department of Neurology and Department of Medical Intensive Care Unit, Chunghua Christian Hospital, Chunghua 500, Taiwan; ^3^Graduate Institute of Chinese Medical Science, China Medical University, Taichung 40402, Taiwan; ^4^Institute of Basic Medical Science, China Medical University, Taichung 40402, Taiwan; ^5^Department of Health and Nutrition Biotechnology, Asia University, Taichung 41354, Taiwan; ^6^Department of Internal Medicine, Chung Shan Medical University Hospital, Taichung 40201, Taiwan; ^7^Institute of Medicine, Chung Shan Medical University, Taichung 40201, Taiwan; ^8^Institute of Biochemistry and Biotechnology, Chung Shan Medical University, Taichung 40201, Taiwan; ^9^Clinical Laboratory, Chung Shan Medical University Hospital, Taichung 40201, Taiwan; ^10^Center for Molecular Medicine, China Medical University Hospital, Taichung 40402, Taiwan; ^11^Department of Biochemistry, School of Medicine, Chung Shan Medical University, Taichung 40201, Taiwan

## Abstract

*Ocimum gratissimum* (OG) is known as a food spice and traditional herb, which has been recommended for the treatment of various diseases. To investigate the hepatoprotective effect of OG aqueous extract (OGAE), male Wistar rats challenged by carbon tetrachloride (CCl_4_) were used as the animal model of chronic hepatic injury. Significantly increased serum catalase and DPPH levels were detected in CCl_4_-administrated rats that were treated with OGAE or silymarin as compared to those rats that were treated with saline or CCl_4_. In contrast, significantly decreased stress proteins including HSP70 and iNOS were observed in livers of CCl_4_-administrated rats that were treated with OGAE or sylimarin as compared to those rats that were treated with saline or CCl_4_. Moreover, significant decreases of MMP-9/MMP-2 ratio, uPA, phosphorylated ERK (p-ERK) and NF-**κ**B (p-P65) were detected in livers of CCl_4_-administrated rats that were treated with OGAE or sylimarin as compared to those rats that were treated with saline or CCl_4_. These findings imply that OGAE can efficiently inhibit CCl_4_-induced liver injuries in rats and may therefore be a potential food or herb for preventing liver injuries.

## 1. Introduction

The liver is the largest organ in human body and necessary for metabolism of drugs and exogenous toxins. Liver damage is a prevalent pathology that involves a variety of disorders including oxidative stress, steatosis, hepatitis, fibrosis, cirrhosis, apoptosis, and hepatocellular carcinoma [1]. However, liver damage due to natural, industrial toxins or drugs is common but rarely recognized [2]. Various xenobiotics are known to cause hepatotoxicity such as carbon tetrachloride (CCl_4_) [3], which alters the antioxidant profile of the liver including superoxide dismutase (SOD), catalase (CAT), glutathione peroxidase (GPx), glutathione reductase (GR), and glutathione transferase (GST) [4]. Moreover, marked increases of serum matrix metalloproteinase (MMP9) [5], aminotransferases, tumor necrosis factor-alpha (TNF-*α*) [6] hepatic HSP70 [7], and inducible nitric oxide synthase (iNOS) protein [6] were detected in animal models after CCl_4_ challenging.

Although a wide range of drugs is currently employed in the management of hepatic disorders, alternative approaches from traditional medicinal systems are increasingly popular in recent days [8]. In traditional systems of medicine, many herbs including *Ocimum *species have been recommended for the treatment of various diseases [9]. The *Ocimum *species*  *are widely found in tropical and subtropical regions and commonly used as food spice and traditional herb. To avoid the side effects by administration of western medicines, growing studies of *Ocimum *species were performed to investigate their therapeutic potentials on hepatic disorders [10–14].

Essential oils obtained from *Ocimum *species showed various medicinal potentials in chemopreventive, anticarcinogenic, free radical scavenging, and radioprotective uses [10–12]. Additionally, ethanolic extract of *Ocimum gratissimum* (OG) leaf also revealed significant chemopreventive effects on chemical-induced papilloma genesis by modulating metabolizing enzymes such as cytochrome P450, glutathione-s-transferase, and aryl hydrocarbon hydroxylase [15, 16]. Moreover, a recent study indicated that administered orally aqueous extract of OG leaf could reduce oxidative and toxicant activity and enhance specific activities of hepatic antioxidant enzymes in rats [13]. Notably, our recent study also indicated that OG leaf aqueous extract (OGAE) may be important in protecting H9c2 cells from H_2_O_2_-induced cell death by inhibiting the mitochondrial dependent apoptosis pathway [14]. Although these studies strongly implicated the medicinal effects of OG, there are only few studies for the beneficial effects of OGAE on chemical-induced hepatic injury. Herein, we investigated the effects of OGAE on reducing hepatic injuries in rats after CCl_4_ challenging.

## 2. Materials and Methods

### 2.1. Preparation of *Ocimum gratissimum* Aqueous Extract and Composition Analysis

Extract of *OG* was prepared as described elsewhere [14, 17]. Briefly, leaves of *OG *Linn were harvested, cleaned with distilled water, and homogenized with distilled water by using polytron. The homogenate was incubated at 95°C for 1 hour (h) and then filtered through two layers of gauze. The filtrate was centrifuged at 20000 g for 15 min at 4°C to remove insoluble pellets, and the supernatant was collected, lyophilized, and stored at −70°C until use. The contents of polyphenol in OGAE were analyzed as indicated in our previous paper [14, 17] and shown in Table 1, revealing the final extract composition of 11.1% polyphenolic acid and 4.5% flavonoids.

### 2.2. Animal Model and Treatments

Thirty-two male Wistar rats (4 weeks old) were obtained from the National Animal Breeding and Research Center, Taipei, Taiwan and acclimatize for 1 week under controlled conditions. The animals were kept under a 12-h light-dark cycle, and ambient temperature was maintained at 25°C. Animals were free access to water and standard laboratory chow (Lab Diet 5001; PMI Nutrition International Inc., Brentwood, MO, USA). Animal welfare and experimental procedures were performed according to the NIH Guide for the Care and Use of Laboratory Animals. All protocols were approved by the Institutional Animal Care and Use Committee of Chung Shan Medical University, Taichung, Taiwan. All the rats were randomly divided into 4 equal groups (8 rats each group). Group I includes control rats injected only liquid paraffin and saline twice a week for 12 weeks (N group); group II includes rats injected intraperitoneal with CCl_4_ (Sigma Chemicals Co. St. Louis, USA) (5 mL kg^−1^ body weight) [18] twice a week for 12 weeks to induce the hepatic injury (E group); group III includes rats injected intra-peritoneal with CCl_4_ as described in group II and administered orally daily with *O. gratissimum* (0.2 mg/kg body weight) [13] for 12 weeks (OGAE group); group IV includes rats injected intraperitoneal with CCl_4_ as described in group II along with silymarin (100 mg/kg) (S group) for 12 weeks. At the end of the experiments, mice were then sacrificed by CO_2_ asphyxiation. The blood samples and liver tissues were collected and stored at −80°C until use.

### 2.3. Catalase Assay (CAT)

The decomposition rate of hydrogen peroxide by CAT was used to assay the enzyme activity according to manufacturer's instruction (EnzyChrom Catalase Assay Kit, BioAssay Systems Co, CA, USA). Briefly, a reaction mixture of 250 *μ*L containing 200 *μ*L of 50 mM phosphate buffer (pH 5.0), 30 *μ*L of 5.9 mM H_2_O_2_, and 10 *μ*L liver supernatant was reacted for one min, and change in absorbance of the reaction solution was measured at 240 nm by a 96-well fluorometric plate reader. CAT activities were expressed as units per milligram of protein (U/mg protein).

### 2.4. DPPH Assay

The radical scavenging activity was determined via a DPPH scavenging activity as described elsewhere [19]. Briefly, 190 *μ*L of 0.1 mM 1,1-diphenyl-2-picrylhydrazyl (DPPH, Sigma Chemicals Co. St. Louis, USA.) solution in ethanol was gently mixed with 10 *μ*L liver supernatant in ethanol. The value of DPPH absorption was measured at 517 nm by a 96-well fluorometric plate reader. DPPH radical scavenging activity was expressed as % inhibition compared to the blank (ethanol).

### 2.5. Western Blot

Sodium dodecyl sulfate-polyacrylamide gel electrophoresis (SDS-PAGE) was performed using 12.5% acrylamide gel. The protein samples were homogenized sufficiently with B25 high-shear dispersing emulsifiers homogenizing machine (BRT CO, Shanghai, China) and centrifuged at 12,000 rpm in 4°C for 30 min. Supernatants were isolated and denatured for 5 min in boiling water with sample buffer (0.0625, M Tris-HCl buffer, pH 6.8, containing 2.3% SDS, 5% 2-mercaptoethanol, and 10% glycerol). Samples applied to the gel were run of 100–150 V for 90 min and electrophoretically transferred to nitrocellulose membrane (Amersham Biosciences, Piscataway, NJ, USA). The membrane was then soaked in PBS with 5% nonfat dry milk for 30 min at room temperature to saturate irrelevant protein binding sites. Antibodies against HSP70, iNOS, uPA, phosphorylated ERK, ERK, NF-*κ*B (p-P65), and *β*-actin (Upstates, Charlottesville, Virginia, USA; Chemicon International, Temecula, CA, USA) were diluted in PBS with 2.5% BSA and incubated for 90 min with gentle agitation at room temperature. The membranes were washed twice with PBS-Tween for 60 min, and secondary antibody conjugated with horseradish peroxidase (HRP) was added for another 30 min. Pierce's Supersignal West Dura HRP Detection Kit (Pierce Biotechnology Inc., Rockford, IL, USA) was used to detect antigen-antibody complexes. The blots were scanned and quantified by densitometry (Appraise, Beckman-Coulter, Brea, CA, USA).

### 2.6. Gel Zymography

MMP-9 and MMP-2 activities were analyzed by gelatin zymography as described elsewhere [20]. Ten microliters of 10× diluted serum or 20 *μ*g protein lysate of livers were separated by an 8% sodium dodecyl sulfate-polyacrylamide gel electrophoresis (SDS-PAGE) gel polymerized with 1 mg/mL gelatin. Gels were washed once for 30 mins in 2.5% Triton X-100 to remove the SDS and then soaked in the reaction buffer containing 50 mM Tris-HCl, 200 mM NaCl, 10 mM CaCl_2_, and 0.02% (w/v) Brij 35 (Sigma, St. Louis, MO, USA; pH 7.5) for 30 mins. The reaction buffer was changed to a fresh one, and the gels were incubated at 37°C for 24 hrs. Gelatinolytic activity was visualized by staining the gels with 0.5% Coomassie brilliant blue and quantified by densitometry (Appraise; Beckman-Coulter, Brea, CA, USA). 

### 2.7. Statistical Analysis

All the statistical analyses were performed using SPSS 10.0 software (SPSS Inc, Chicago, IL, USA). Three independent experiments were repeated. Statistical analyses were performed using the Student's *t* test or one-way ANOVA. *P* < 0.05 was considered statistically significant.

## 3. Results

### 3.1. OGAE Increases the Serum Catalase and DPPH Levels in Serum of Rats Treated with CCl_4_

**Figure 1:**
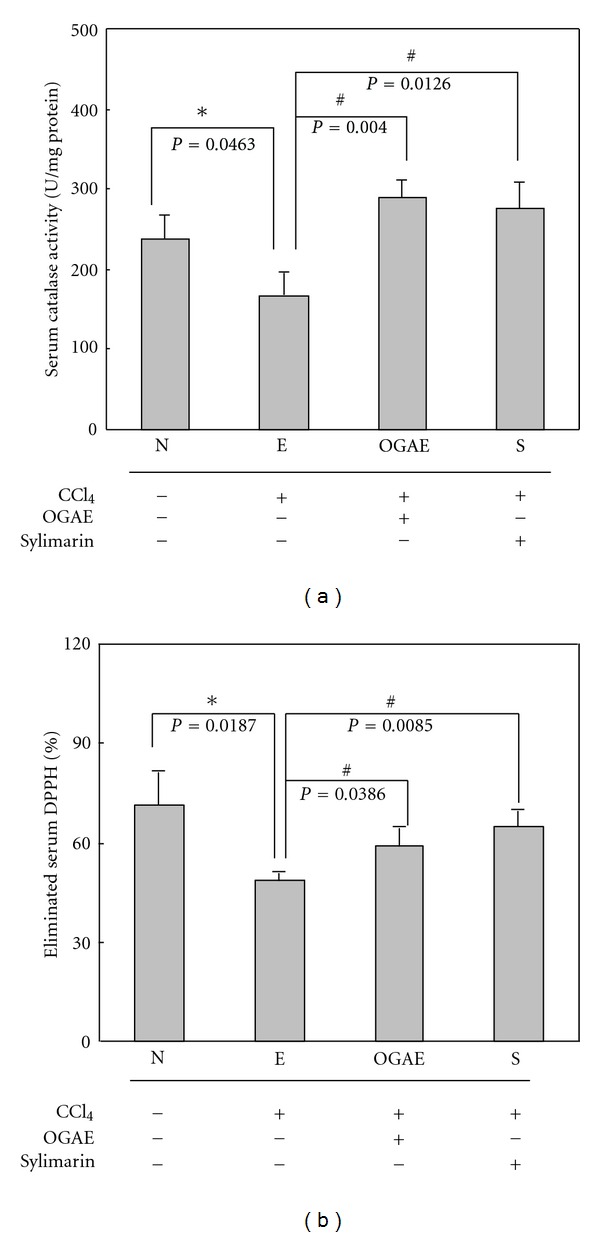
Detection of (a) catalase activity and (b) eliminated DPPH in serum of rats with different treatment. The average result ± SE of three independent experiments is shown. ∗ and # indicate significant difference, *P* < 0.05.

To examine the effects of OGAE on antioxidant activities, the levels of catalase and DPPH in serum of rats with different treatment were examined. Significant decreases of serum catalase and DPPH were detected in rats treated with CCl_4_ (E group) as compared to those rats from control group (Figures 1(a) and 1(b)). In contrast, significant increases of catalase and DPPH levels were observed in serum of rats from OGAE and S groups as compared to those rats from E group (Figures 1(a) and 1(b)).

### 3.2. OGAE Decreases the Expressions of HSP70 and iNOS Proteins in Livers of Rats Treated with CCl_4_

**Figure 2:**
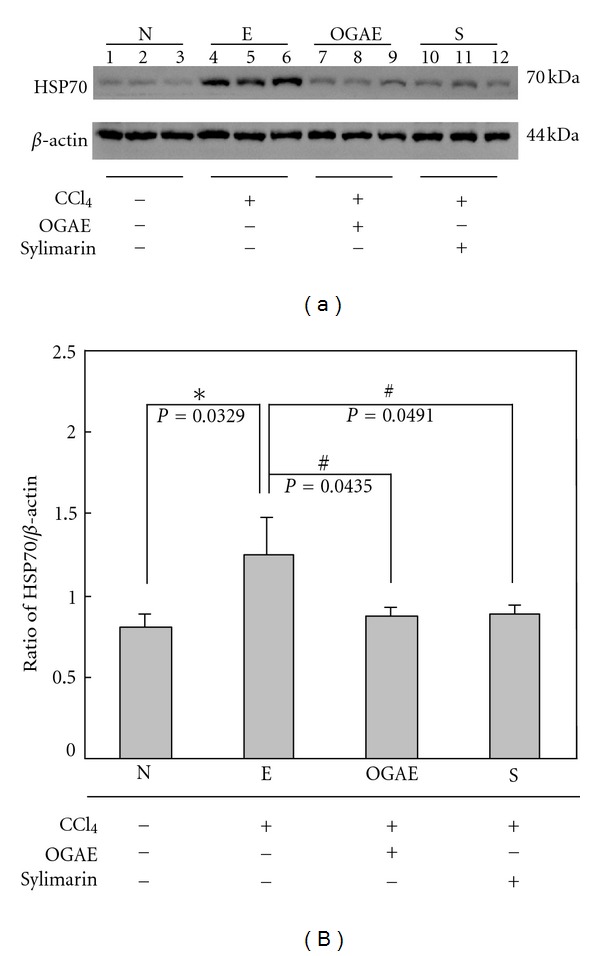
The levels of (a) HSP70 protein in livers from rats with different treatment were detected by Western blotting. (b) Bars represent the relative protein quantification of HSP70 on the basis of *β*-actin. Similar results were observed in three independent experiments. ∗ and # indicate significant difference, *P* < 0.05.

**Figure 3:**
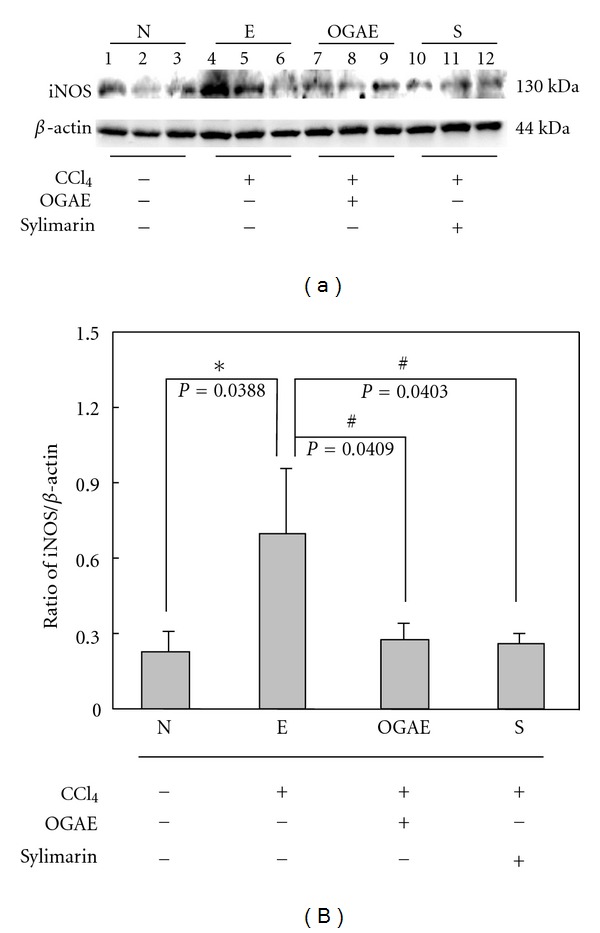
The levels of (a) iNOS protein levels in livers from rats with different treatment were detected by Western blotting. (b) Bars represent the relative protein quantification of iNOS on the basis of *β*-actin. Similar results were observed in three independent experiments. ∗ and # indicate significant difference, *P* < 0.05.

To examine the effects of OGAE on stress proteins after CCl_4_ challenging, Western blots were performed to detect the expressions of HSP70 and iNOS proteins in livers of rats with different treatments. Significant increases of HSP70 were detected in livers of rats from E group as compared to those rats from N group (Figure 2(a)). However, significant decreases of HSP70 proteins were observed in livers of rats from both OGAE and S groups as compared to those rats from E group (Figure 2(a)). Quantified results were shown in Figure 2(b). In addition, similar results were observed in iNOS expression. Significant increases of iNOS proteins were detected in livers of rats from E group as compared to those rats from control group (Figure 3(a)). In contrast, significant decreases of iNOS proteins were detected in livers of rats from both OGAE and S groups as compared to those rats from E group (Figure 3(a)). Quantified results were shown in Figure 3(b).

### 3.3. OGAE Reduces MMP-9 Activity and uPA Protein Expression through Inhibiting ERK and NF-*κ*B Signaling in Rats Treated with CCl_4_

**Figure 4:**
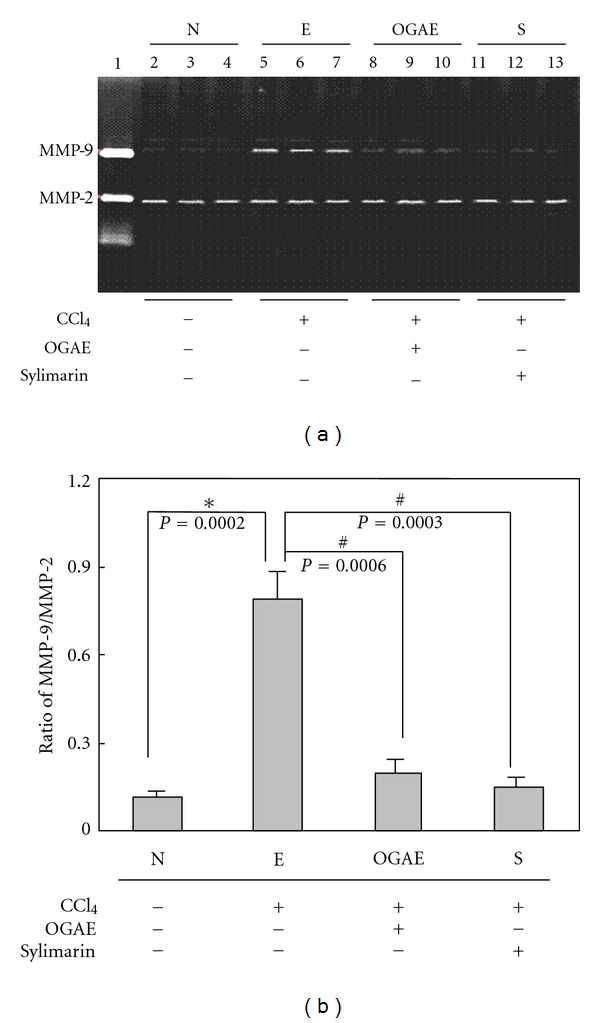
Detection of (a) MMP-9 and MMP-2 activities in livers of rats with different treatment by gel zymography. (b) Signal intensity was quantitated using a Phosphoimager, and the ratio of MMP-9/MMP-2 was presented. Similar results were observed in three independent experiments. ∗ and # indicate significant difference, *P* < 0.05.

**Figure 5:**
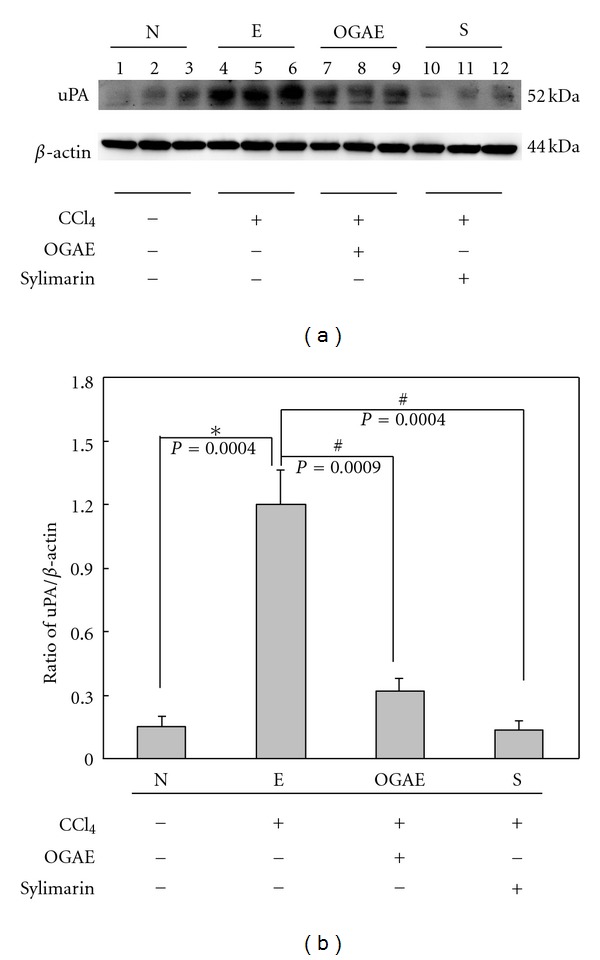
The levels of (a) uPA protein levels in livers from rats with different treatment were detected by Western blotting. (b) Bars represent the relative protein quantification of uPA on the basis of *β*-actin. Similar results were observed in three independent experiments. ∗ and # indicate significant difference, *P* < 0.05.

**Figure 6:**
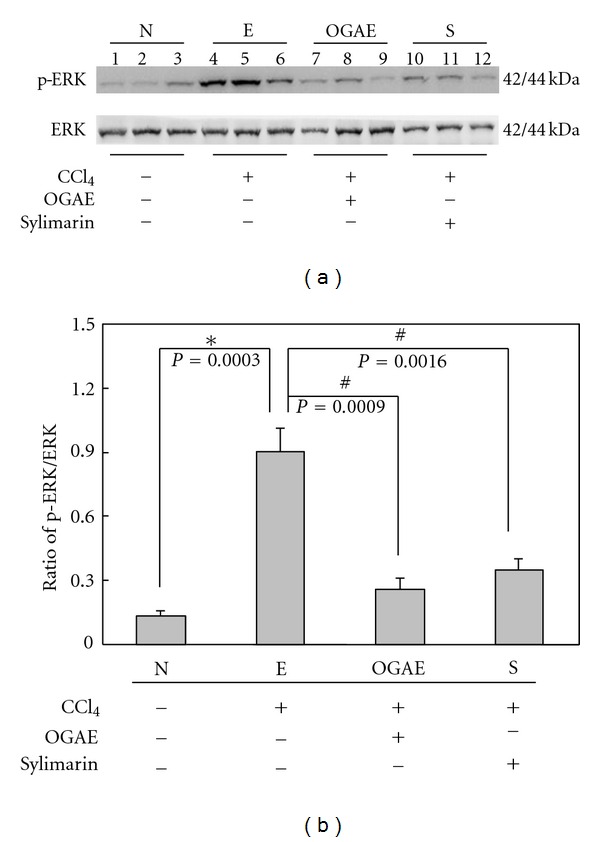
The levels of (a) phosphorylated ERK and ERK protein levels in livers from rats with different treatment were detected by Western blotting. (b) Bars represent the relative protein quantification of phosphorylated ERK on the basis of ERK. Similar results were observed in three independent experiments. ∗ and # indicate significant difference, *P* < 0.05.

**Figure 7:**
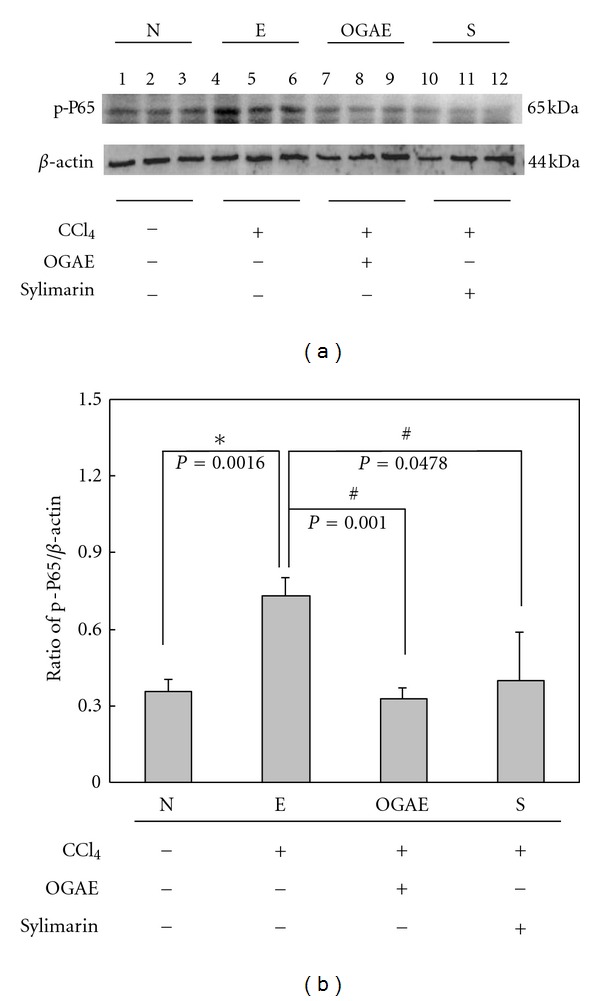
The levels of (a) phosphorylated P65 and P65 protein levels in livers from rats with different treatment were detected by Western blotting. (b) Bars represent the relative protein quantification of phosphorylated P65 on the basis of P65. Similar results were observed in three independent experiments. ∗ and # indicate significant difference, *P* < 0.05.

MMP-9 is known as an indicator playing important roles in hepatic disorders. To investigate the effects of OGAE on MMP-9, gel zymography was performed to detect the MMP-9 activity. Significant increase of MMP-9/MMP-2 ratio was detected in liver of rats from E group as compared to those rats from N group (Figure 4(a)). Notably, significant decreases of MMP-9/MMP-2 ratio were observed in livers of rats from both OGAE and S groups (Figure 4(a)). Quantified results were shown in Figure 4(b). In addition, the expression of uPA protein, an upstream activator of MMP-9, was also examined by Western blot. As shown in Figure 5(a), significant increase of uPA protein was observed in liver of rats from E group as compared to those rats from N group (Figure 5(a)). In contrast, significant decreases of MMP-9/MMP-2 ratio were observed in livers of rats from both OGAE and S groups as compared to those rats from E group (Figure 5(a)). Quantified results were shown in the lower panel of Figure 5(b). To further investigate the influence of OGAE on MMP-9 signaling, presence of ERK protein and its phosphorylated form were examined. Significantly increased ratio of p-ERK/ERK was detected in liver of rats from E group as compared to those rats from N group (Figure 6(a)). In contrast, significant decreases of p-ERK/ERK ratio were observed in livers of rats from both OGAE and S groups as compared to those rats from E group (Figure 6(a)). Quantified results were shown in Figure 6(b). Moreover, similar results were observed in phosphorylation of NF-*κ*B (p-P65). Significantly increased ratio of p-65/*β*-actin ratio was detected in liver of rats from E group as compared to those rats from N group (Figure 7(a)) whereas significant decreases of p-65/*β*-actin ratio were observed in livers of rats from both OGAE and S groups as compared to those rats from E group (Figure 7(a)). Quantified results were shown in Figure 7(b). 

## 4. Discussion

Although growing evidences have indicated the therapeutic potentials of *Ocimum *species on hepatic disorders, only few studies for the therapeutic effects of OGAE on chemical-induced hepatic injury were performed. In current study, we further reported the beneficial effects of OGAE on increasing serum catalase and DPPH levels and reducing hepatic HSP70 and iNOS protein in livers of CCl_4_-administrated rats. In the meantime, we found that OGAE also reduces the ratio of MMP-9/MMP-2, uPA protein level via ERK, and NF-*κ*B phosphorylation signaling.

Liver injury induced by CCl_4_ is a well-known experimental model [21–23]. The hepatic toxicity of CCl_4_ is mainly through the generation of trichloromethyl free radical in liver microsomes and consequently induces lipid peroxidation [24]. Marked reduction of antioxidant levels was observed in animal models after CCl_4_ challenging, including SOD, CAT, GPx, GR, and GST [4]. Meanwhile, various hepatic damaged markers such as HSP70 and iNOS were also elevated [6, 7]. In current study, our experimental results indicated that OGAE and silymarin exhibited a significant hepatoprotective effect as evident from the increase of serum CAT and DPPH as compared with control group. In addition, significantly reduced hepatic damaged markers, HSP70 and iNOS, were detected in both OGAE or silymarin-treated rats as compared to control group. These findings implied the potentials of OGAE on increasing antioxidant activity and reducing inflammatory associated proteins in livers of rats after CCl_4_ challenging as well as the silymarin does.

MMP9 is a member of the MMP protein family and plays a crucial role in various hepatic disorders [25], including inflammatory processes [26], fibrogenesis [27], and cancers [28, 29]. A variety of studies have indicated that CCl_4_ could induce hepatic injury via elevating MMP9 protein level and activity [30–33]. In addition, urokinase-type plasminogen activator (uPA) has been demonstrated to upregulate MMP-9 expression in both gene transcription and protein synthesis [34]. Decreased expression of MMPs by inhibiting the uPA system could provide the microvascular protection in animal model of cerebral ischemic rats [35]. Moreover, ERK1/2 and NF-*κ*B signaling is known to play crucial roles in up-regulation of MMP9 [29, 36]. These studies suggested the importance of MMP9 signaling in liver injuries. Notably, our experimental results exhibited a significant hepatoprotective effect of OGAE by decreasing MMP9, uPA, p-ERK/ERK ratio, and phosphorylated P65 in livers of CCl_4_-challenging rats as compared to controls.

**Figure 8:**
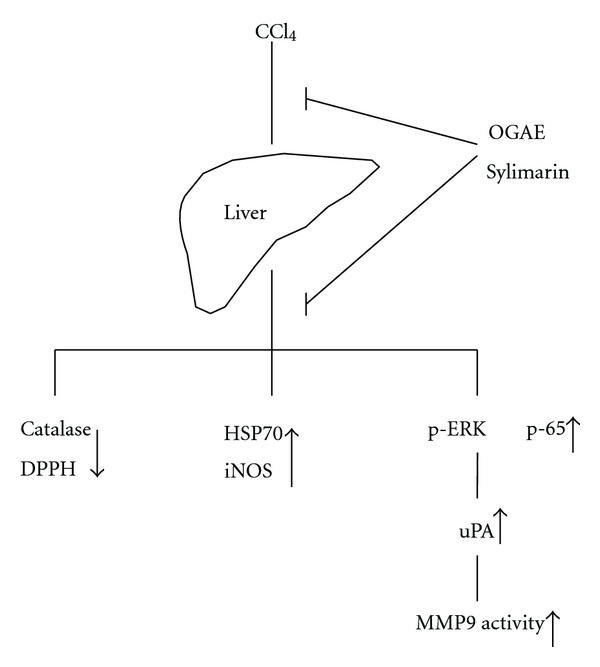
Proposed model for the inhibitory effects of *Ocimum gratissimum* aqueous extract and silymarin on the CCl_4_-induced hepatic stress in rats. Our data demonstrated that *Ocimum gratissimum* aqueous extract revealed very similar effects on hepatic protection as silymarin by increasing antioxidant activities, reducing of stress-related proteins and MMP9 activity through ERK and NF-*κ*B signaling in livers from CCl_4_-challenging rats. OGAE indicates *Ocimum gratissimum* aqueous extract.

Polyphenols from plant extracts have been indicated as being major therapeutic components for oxidative stress. Although the cellular mechanisms underlying the actions of polyphenols and their metabolites have not been completely interpreted, it is believed that their properties including antioxidant activity, free radical scavenging, and anti-inflammation should be involved [37]. Silymarin is known as a purified extract from *Silybum marianum* (L.) Gaertn and composed of silibinin, isosilibinin, silydianin, and silychristin. This extract has been wildly used as a remedy for nearly 2000 years and remains being used as a medicine for many types of acute and chronic liver diseases [38, 39]. However, various side effects of siymarin such as nausea, mild headache, diarrhea, vomiting, and joint pain were reported [38, 40]. Recently, similar components of polyphenols and effects on hepatic protection were reported in studies of *O. gratissimum* [11–14]. As illustrated in Figure 8, our results revealed that both silymarin and OGAE have very similar effects on hepatic protection by increasing antioxidant activities, reducing stress-related proteins and MMP9 activity through ERK and NF-*κ*B signaling in CCl_4_-challenging rats. Additionally, the beneficial effects of OGAE were observed along with the administration of CCl_4_ in this study. Therefore, we consider that the potential benefit of OGAE should be preventive or neutralizing on CCl_4_-induced acute liver injury rather than therapeutic. However, further study is merited to investigate whether OGAE has therapeutic effects on CCL_4_-induced liver injury.

Altogether, the current study shows that OGAE supplement to CCl_4_-administrated rats leads to several beneficial alternations at multiple levels in livers and suggest the potential of OGAE in protective application. Although silymarin and OGAE share the similar effect and effectiveness in terms of anti-injury of liver caused by CCl_4_; herein we indeed provide another possible health food or alternative medicine for alleviating acute liver injuries.

## Figures and Tables

**Table 1:** Major components of phenolic acids and flavonoids in OGAE.

Ingredients	Concentration (mg/g)	Percentage (%)
Epicatechin	3.7	0.37
Caffeic acid	2.7	0.27
Rutin	2.5	0.25
Catechin	0.3	0.03
Gallic acid	n.d.	—
Protocatechuic acid	n.d.	—
Epigallocatechin gallate	n.d.	—
Quercetin	n.d.	—
Naringenin	n.d.	—

n.d: not determined.

OGAE indicates *ocimum gratissimum* aqueous extract.
